# FSGS: Diagnosis and Diagnostic Work-Up

**DOI:** 10.1155/2016/4632768

**Published:** 2016-05-24

**Authors:** Ben Sprangers, Björn Meijers, Gerald Appel

**Affiliations:** ^1^Department of Microbiology & Immunology, KU Leuven, 3000 Leuven, Belgium; ^2^Department of Nephrology, University Hospitals Leuven, 3000 Leuven, Belgium; ^3^Center for Glomerular Diseases, Columbia University Medical Center, New York, NY 10032, USA

## Abstract

Focal segmental glomerulosclerosis is a histologic lesion, rather than a clinical disease. FSGS is common cause of nephrotic syndrome in both adults and children worldwide. In the United States it is the most common primary glomerular disease resulting in end-stage renal disease and recent reports have suggested that its incidence might be on the rise. Currently the incidence is estimated to be 7 per million. The podocyte is the cellular target cell in FSGS and in recent years substantial insight in the pathogenesis and genetics of FSGS have accumulated. Furthermore the discovery of potential novel biomarkers to diagnose FSGS and monitor disease activity has renewed interest in this disease. In this review article we will focus on the clinical presentation and diagnosis of FSGS.

## 1. Introduction

FSGS is estimated to be responsible for 40% of adult nephrotic syndromes and 20% of pediatric nephrotic syndromes and has an incidence of 7 per million [1]. FSGS has an estimated prevalence of 4% and is the most common primary glomerular disease resulting in end-stage renal disease in the United States [2]. FSGS is a histologic lesion resulting from glomerular injury primarily affecting the podocytes and characterized by the presence of sclerosis in parts (segmental) of some (focal) glomeruli. Initially lesions are confined to a limited number of glomeruli and are segmental in nature. FSGS is commonly termed primary or secondary. Primary FSGS is caused by a primary podocytopathy while secondary FSGS denotes the development of FSGS lesions as an adaptive phenomenon following a reduction in nephron mass, direct toxicity by drugs or viral infections, or healing from endothelial injury. Upon identification of FSGS in a renal biopsy a thorough examination should be initiated to identify the underlying cause. The distinction between primary and secondary FSGS is important for both prognostic and therapeutic reasons.

## 2. Epidemiology

In the United States FSGS is the most common histologic lesion found in patients with adult nephrotic syndrome accounting for 35% of all cases and even 50% of cases among individuals from African American descent [3]. The frequency of FSGS as cause of adult nephrotic syndrome in African Americans is 2-3 times higher compared to Caucasian individuals [3]. In the time period 1975 to 1994, FSGS accounted for 57% of cases in blacks (versus 23% in whites), and its prevalence in blacks increased from 39% to 64% in 1975–1984 and 1985–1994, respectively [4]. Furthermore, African Americans with FSGS have a higher likelihood of a family history of ESRD [5]. The high incidence of FSGS in African Americans with the nephrotic syndrome and the genetic predisposition to this lesion in blacks (vide infra) explains in part why reported incidences in other countries are considerable lower. For example, in a 2004 Spanish study, membranous nephropathy (24%) was the most common cause of nephrotic syndrome in patients between 15 and 65 years with FSGS only being the 4th most common cause (12%) [6]. However, a number of studies show the incidence of FSGS is increasing in non-blacks in both Canada and the United States [7] (Dan Cattran, personal communication). A study of the United Renal Database System demonstrated that idiopathic FSGS is the most common primary glomerular disease resulting in end-stage renal disease in both black and white people in the United States [2]. Data suggest that FSGS as cause of ESRD is on the rise from 0.2% in 1980 to 2.3% in 2000 with African Americans at an increased risk [2].

## 3. Clinical Presentation

Proteinuria is the most common presenting feature of FSGS. In patients with primary FSGS the full nephrotic syndrome is very common and often associated with hypertension, microscopic hematuria, and some degree of renal insufficiency [8, 9]. In secondary FSGS, patients tend to have subnephrotic-range proteinuria at presentation (although nephrotic range proteinuria will develop in the majority over time) in the absence of oedema, hypoproteinaemia, or hypoalbuminaemia [10, 11].

FSGS can be classified according to etiology into the following (see Table 1).

Primary or idiopathic FSGS are undefined circulating factors that mediate abnormal glomerular permeability with podocyte injury and dedifferentiation.

Secondary FSGS are as follows:

(1) Familial/genetic:
   cfr (Table 1).
(2) Virus-associated:
   HIV, parvo B19, CMV, EBV, hepatitis C, and Simian virus 40.
(3) Drug-induced:
   heroin, interferon, lithium, pamidronate, sirolimus, and anabolic steroid.
(4) Mediated by adaptive structural-functional responses to the following:
   
*✓* reduced renal mass with hyperfiltration and stretch on podocytes:
     oligomeganephronia,
     unilateral renal agenesis or hypoplasia,
     renal dysplasia,
     reflux interstitial nephropathy,
     surgical renal ablation,
     obesity,
     low birth weight,
     chronic allograft nephropathy,
     extensive loss of functional nephrons secondary to any advanced renal disease.
   
*✓* Ischemia:
     (malignant) hypertension,
     cholesterol crystal emboli,
     renal artery stenosis,
     atheroemboli or other acute vasoocclusive processes,
     hypertensive nephrosclerosis,
     cyanotic congenital heart disease,
     renal transplant rejection,
     calcineurin-inhibitor toxicity.

FSGS can histologically be subdivided according to the Columbia classification into the following:

- Classical FSGS or FSGS NOS (not otherwise specified).
- Collapsing variant (although there is discussion whether this is truly FSGS or rather a distinct pathology).
- Tip variant.
- Perihilar variant.
- Cellular variant.

About 80% of cases are primary or idiopathic. FSGS is by some authors believed to be closely related to minimal change disease and both diseases are postulated to be part of the same spectrum of diseases (podocytopathies) [12]. Injury of the podocytes gives rise to foot process effacement and proteinuria. The distinction between primary and secondary FSGS can be made on both clinical and histologic bases. Nephrotic range proteinuria without the full nephrotic syndrome is suggestive for secondary FSGS. Secondary FSGS is usually associated with slowly increasing proteinuria (initially subnephrotic) [10, 11], lower prevalence of nephrotic syndrome, higher serum albumin, lower serum cholesterol, lower rate of edema, and progressive renal insufficiency over time [10, 13, 14]. Interestingly, despite massive proteinuria most patients with secondary FSGS (due to obesity, reflux nephropathy, or renal mass reduction) usually do not develop full blown nephrotic syndrome [13]. The precise reason for this dichotomy is not clear but has been suggested to be related to the development of secondary compensatory mechanism during a more gradual appearance of proteinuria in secondary FSGS [13]. Another histologic variant where massive proteinuria may not be associated with the development of edema is the collapsing FSGS variant. This is, however, believed to be related to accompanying rapid loss of GFR [15–17]. The distinction between primary and secondary FSGS has important therapeutic implications as primary FSGS often requires immunomodulatory treatment whereas treatment in secondary FSGS should be focused on the reduction of intraglomerular hypertension using RAAS blockade.

In infants with steroid-resistant nephrotic syndrome, a genetic defect can be identified in up to two-thirds of patients [18]. Furthermore, genetic testing is more likely to identify a genetic basis of FSGS in young children, patients with syndromic disease, or a positive family history. In a Spanish study in patients with steroid-resistant nephrotic syndrome, the percentage of patients in whom a genetic basis could be identified in congenital-onset, infantile-onset, early and late childhood onset, adolescent-onset, and adult-onset was 100, 57, 24 and 36, 25, and 14%, respectively [19]. A study of the PodoNet Consortium found a 11% overall percentage of disease-causing abnormalities in adolescent with steroid-resistant nephrotic syndrome [20].

Histologically FSGS is classified in 5 different subtypes: perihilar, tip, collapsing, cellular, and not otherwise specified [21]. A recent study evaluated renal biopsies of 138 patients included in the FSGS Clinical Trial to study the association between histologic subtype and clinical features and outcome [22]. The histologic subtype was associated with clinical features: patients with NOS FSGS were more likely to present with subnephrotic proteinuria whereas patients with tip or collapsing variants tended to be older and have higher degrees of proteinuria and hypoalbuminemia at presentation [22]. Tip variant in this study was associated with Caucasian race, lower baseline creatinine, and rate of progression. In contrast, the collapsing variant was associated with African American descent, elevated baseline creatinine, and higher rate of progression [22]. The perihilar form is common in secondary FSGS in patients with obesity, hypertension, reflux nephropathy, or renal agenesis, and patients usually have subnephrotic proteinuria. The tip form is usually primary presenting with sudden onset of nephrotic syndrome and has a good prognosis (high response rate to glucocorticoids and low risk of progression). The cellular variant can be both primary or secondary and is the least common variant usually presenting with a nephrotic syndrome. The NOS can also be primary or secondary and is the most common variant. The collapsing subtype can be both primary or secondary and has an infaust prognosis (severe nephrotic syndrome resistant to immunosuppressive treatment and associated with rapid progression to renal failure).

Both clinical and histologic features have been reported to be predictive towards outcome. African American descent, degree of proteinuria, and renal insufficiency have been associated with poor outcome. Chun et al. have reported that the attainment of partial of complete remission is associated with better long-term outcome in primary adult FSGS [8]. As histology is concerned, increased degrees of interstitial fibrosis and tubular atrophy are a predictor of poor outcome.

## 4. Diagnostic Work-Up

When confronted with a patient with FSGS a careful medical history and clinical examination should be performed. The presentation (sudden onset of nephrotic syndrome or more subtle gradual changes) and associated medical conditions (infections, obesity, hypertension, etc.) help to make a distinction between primary and secondary FSGS. Also careful attention should be paid to the medications and drugs the patient is using. Lab testing including viral serology, kidney function, serum albumin, and lipids should be performed. A biopsy is necessary to establish the diagnosis of FSGS and determine the subtype of FSGS. Genetic screening of patients with steroid-resistant nephrotic syndrome should be performed when FSGS occurs early in life as the likelihood of identifying a genetic basis for FSGS is high. Genetic screening of adolescent/adult patients with FSGS can be done relatively quickly these days but its place in clinical practice is not clear at this moment. The interpretation of negative results especially remains problematic as these patients may have mutation in noncoding regions of candidate genes or mutations in novel genes not yet reported. Undoubtedly at this time there are many more genetic causes FSGS to be discovered. Exome sequencing is expected to dramatically improve our knowledge in this respect; however, exome sequencing requires specialized bioinformatics support for analysis. It has been suggested by some authors to screen also adolescents and adults with steroid-resistant nephrotic syndrome to avoid unnecessary exposure to second-line immunosuppressive therapies [20]. However, the therapeutic implications of results from genetic screening as some patients with WT1-mutation associated steroid-resistant nephrotic syndrome have been reported to have a favorable response to cyclosporine [23]. In the setting of transplantation, genetic testing could be useful as it has been demonstrated that patients with inherited forms of FSGS have a low risk of recurrence after transplantation [24, 25].

## 5. Histology

Initially FSGS lesions are concentrated in the corticomedullary region and are therefore easily missed on biopsy. Cellular changes within the podocyte precede scarring as the initial event will affect the podocyte attachment to the glomerular basement membrane. Denudation of the glomerular basement membrane will result in sticking of capillary loops and collapse. Subsequently, sclerosis, deposition of hyaline material, adhesions to Bowman's capsule, and synechiae formation will develop. Even segmental lesions will result in glomerular dysfunction due to misdirected filtration and filtrate spreading on the remaining part of the nephron [26]. FSGS is probable not as focal and segmental as suggested by its name. Some subtypes of FSGS the histologic lesions even are not focal, segmental, or sclerotic, that is, glomerular tip lesion and collapsing glomerulopathy. In animal models of FSGS almost all glomeruli show sclerotic lesions on three-dimensional morphometric analysis [27]. As the volume of the sclerotic lesion is on average only 12.5% of the total glomerular volume, the number of glomeruli affected by sclerosis is grossly underestimated on conventional single section kidney biopsy evaluation [28]. As a consequence, a kidney biopsy containing only few glomeruli cannot exclude FSGS. It is to be advised that consecutive sections are evaluated from 12 to 15 serial sections and at least 8 glomeruli are analyzed [21, 28]. Furthermore, initial changes of FSGS can be limited and only detectable by electron microscopic examination. Therefore, it is not uncommon that after an initial biopsy showing no clear FSGS lesion a subsequent biopsy taken months or years later shows clear FSGS lesions [29]. With progression of the disease more widespread and global glomerulosclerosis develops together with tubulointerstitial and vascular lesions.

Histologically FSGS is classified in 5 different subtypes: perihilar, tip, collapsing, cellular, and not otherwise specified [21]. A study from the Columbia group demonstrated that the most common FSGS variant in all FSGS patients was NOS or perihilar variant (62.3%) followed by collapsing (23.7%), tip (9.4%), and cellular (3%) variants [30]. Another study reported similar data: NOS, 42%; perihilar, 26%; collapsing, 11%; tip, 17%; and cellular, 3% [31]. In the FSGS Clinical Trial the incidences in children and young adults with steroid-resistant FSGS were 68% in FSGS not otherwise specified, 12% in collapsing, 10% in tip, 7% in perihilar, and 3% in cellular variants [22, 32]. The rates of complete and partial remission have been shown to be related to the histologic subtype: with the highest remission rates for tip variant, intermediate remission rate for cellular, perihilar, and NOS variants, and the lowest remission rates for the collapsing variant. Moreover, several reports have demonstrated that the histologic variant of FSGS is independently associated with outcome [30, 31]; in an analysis of the FSGS Clinical Trial in which patients between 2 and 40 years old with steroid-refractory primary FSGS were randomly assigned to receive either cyclosporine or dexamethasone plus MMF the risk of ESRD at 3-year follow-up was 47% in collapsing, 20% in not otherwise specified, and 7% in tip variant patients [22]. In general, the outcome for secondary FSGS is better than that for primary forms as a consequence of increased likelihood to obtain remission with RAAS blockade and lower serum creatinine at presentation [11].

Histologically secondary FSGS is predominantly associated with the perihilar variant and limited foot process effacement confined to sclerotic areas, whereas foot process effacement is diffuse in primary FSGS [10, 12]. Foot process effacement is most severe in cases of primary FSGS while being relatively limited in secondary forms of FSGS and there is little overlap between them [33]. As the variants of primary FSGS are concerned: foot process effacement is more pronounced in tip, cellular, and collapsing variants, while it is variable in the not otherwise specified variant and limited in the perihilar variant [12]. Some forms of secondary FSGS are associated with widespread foot process effacement: HIV-associated FSGS [16] and FSGS associated with use of interferon [34] and pamidronate [35].

## 6. Biomarkers

SuPAR has been proposed to be a new marker to diagnose and predict FSGS recurrence after transplantation and monitor treatment response in patients with primary FSGS [36–38]. The findings of the initial paper by Chicago group could not be confirmed by other groups and an important inverse relationship between suPAR and eGFR has become evident [39–45]. Moreover, urinary suPAR has been proposed as a marker as well [46]. Thus, although suPAR may be a marker for progressive renal damage [47], it cannot be considered a biomarker for FSGS. More recently, it has been suggested that podocyte expression of B7-1 (CD80) may help to differentiate between primary and secondary FSGS. CD80 is under normal condition not expressed on human podocytes and has been shown to be upregulated in patients with MCD and primary FSGS [48]. A recent report suggested that CD80 expression on podocytes could differentiate primary from secondary FSGS and predict response to abatacept [49]. Urinary CD80, however, appears to be elevated in minimal change disease (especially minimal change disease in relapse) and not in FSGS [50–52]. Moreover, other groups have either not been able to stain FSGS podocytes for B7-1 or, when able to stain, not been able to show a response to costimulatory blockade as seen with abatacept [53, 54]. Recently positive B7-1 staining of podocytes has been found in one group in both animal models and patients with diabetes mellitus [55]. Again other groups have not been able to confirm these results [56]. Thus, until there is consistent and reproducible staining techniques for this biomarker, there will be no clinical test for FSGS using it.

The detection of activated parietal epithelial cells immunohistochemically has been shown to be able to make a distinction between early FSGS and minimal change disease [57] and show similar patterns in both primary and secondary FSGS [58]. FSGS lesions can also be associated with rare genetic diseases (Dent's disease) [59] and tubulopathies [60]. Concerning tubulopathies causing FSGS lesions it was suggested by Sethi and colleagues to include a comparison of urinary protein/creatinine ratio to a urinary albumin/creatinine ratio in the diagnostic work-up of FSGS [61]. If <40–50% of total proteinuria is the result of albuminuria the possibility of tubular proteinuria or the presence of light chains should be excluded.

## 7. Genetic Testing

Several genes, instrumental in podocyte homeostasis, have been reported to be associated with genetic forms of FSGS and these findings have propelled the field of podocyte biology in the last decade (Table 1) [62]. Numerous genetic defects have been associated with FSGS [63] and these genetic forms account for a significant proportion of patient with steroid-resistant disease in young children [64, 65]. Most of the genetic defects are located in genes coding for proteins involved in glomerular basement membrane formation and/or podocyte biology. Both autosomal dominant and autosomal recessive inheritance have been described. As penetrance is not 100% the detection of familial or genetic FSGS can be difficult. A family history and onset in early life are suggestive for genetic forms of FSGS. Histology does not allow for the differentiation between genetic and nongenetic forms of FSGS except in NPHS1 and alpha-actinin-4 mutations. The usefulness of genetic testing in the setting is disputed and is dependent on the presence of a familial history and onset in early life [19, 66]. In the first year of life the most common causes of genetic FSGS are mutations in nephrin and podocin genes which present in an autosomal recessive manner and with a full blown nephrotic syndrome. In contrast, during adolescence and early adulthood most cases of genetic FSGS are caused by autosomal dominant forms caused by mutations in TRPC6 and alpha-actinin-4 [18, 19]. Genetic abnormalities in the inverted formin 2 gene may be one of the more common forms of hereditary FSGS [67]. FSGS in adulthood is rarely caused by a genetic abnormality and often proteinuria is not massive [19]. Commercial tests are currently available to detect NPHS1 and NPHS2 mutations but not the alpha-actinin-4, TRPC6, or CD2AP genes.

It is well established that FSGS is more prevalent and the course of disease is more severe as well in African American individuals. Besides socioeconomic factors, genetic factors such as variants in the apolipoprotein 1 gene are closely related to the development of nondiabetic kidney diseases in African Americans. Initial studies pointed to variants in the MYH9 gene (which is located closely to the APOL1 gene) as risk factor for kidney disease in African Americans [68, 69]. Subsequent studies showed that, however, a strong linkage disequilibrium exists in the chromosomal region of APOL1 and MYH9, and it is now accepted that the MYH9 haplotype simply reflects APOL1 variation [70]. APOL1 risk variants are associated with 17-fold higher odds for FSGS and 29-fold higher odds for HIV-associated nephropathy [71]. In recent studies, APOL1 risk genotype was determined in a subset of patients included in the FSGS Clinical Trial [72]. The 2 risk alleles were predominantly present in African American patients and were associated with collapsing variant and an increased risk of progression to ESRD. Interestingly, APOL1 risk alleles did not influence the response to treatment [72]. These APOL1 risk alleles for kidney disease have been associated with resistance to African trypanosomiasis and are geographically restricted [70, 73, 74]. There is an ongoing discussion whether African American individuals with subnephrotic-range proteinuria, with an FSGS lesion on biopsy associated with only segmental foot process effacement, and with the APOL1 risk alleles should receive a diagnosis of FSGS or should alternatively by diagnosed with APOL1-associated nephropathy.

## 8. Conclusion

Focal segmental glomerulosclerosis is a histologic lesion, rather than a clinical disease. FSGS is common cause of nephrotic syndrome in both adults and children worldwide. FSGS is divided into primary and secondary FSGS. This distinction is mainly important for therapeutic reasons. The podocyte is the cellular target cell in FSGS and in recent years substantial insight in the pathogenesis and genetics of FSGS have accumulated. Furthermore the discovery of potential novel biomarkers to diagnose FSGS and monitor disease activity has renewed interest in this disease.

## Figures and Tables

**Table 1:** Genetic defects associated with FSGS.

	Gene	Inheritance	Location	Function of the encoded protein
*Slit diaphragm proteins*				
(i) Nephrin	NPHS1	AR	19q13.1	Member of the immunoglobulin family, cell adhesion molecules
(ii) Podocin	NPHS2	AR	1q25 31	Regulation of glomerular permeability
(iii) CD2 associated protein	CD2AP	AD (AR)	6p12	Scaffolding molecule that regulates the actin cytoskeleton

*Cell membrane associated proteins*				
(i) Transient receptor potential cation channel 6	TRPC6	AD	11q21 22	Receptor-activated calcium channel in the cell membrane
(ii) Protein tyrosine phosphatase receptor type O	PTPRO	AR	12p22	Member of the R3 subtype family of protein tyrosine phosphatases at the apical surface of polarized cells
(iii) Laminin 2	LAMB2	AR	3p21	Family of extracellular matrix glycoproteins in the basement membranes
(iv) 4 integrin	ITGB4	AR	17q11	Transmembrane glycoprotein receptors
(v) Tetraspanin CD151	CD151	AR	11p15	Member of the transmembrane 4 superfamily, cell-surface proteins
(vi) LIM homeobox transcription factor 1*β*	LMX1B	AD	9q33.3	Member of LIM-homeodomain family, transcription factor

*Cytosolic or cytoskeletal proteins*				
(i) Actinin 4	ACTN4	AD	19q13	Member of the spectrin gene superfamily, cytoskeletal proteins
(ii) Phospholipase C1	PLCE1	AR	10q23 24	Member of the apolipoprotein C1 family, role in HDL and VLDL metabolism
(iii) Myosin heavy chain 9	MYH9	AD	22q12.3	Nonmuscle myosin, involved in cytokinesis, cell motility, and maintenance of cell shape
(iv) Inverted formin 2	INF2	AD	14q32	Member of the formin family, function in de- and polymerization of actin filaments
(v) Myosin 1E	MYO1E	AR	15q21 26	Member of the nonmuscle class I myosins, involved in intracellular movement and membrane trafficking
(vi) Rho GDP-dissociation inhibitor 1	ARHGDIA	AR	17q25	Key role in the regulation of signaling through Rho GTPases

*Nuclear proteins*				
(i) Wilms tumor 1	WT1	AD	11p13	Transcription factor, role in the normal development of the urogenital system
(ii) SMARCA-like protein	SMARCAL1	AR	2q34 36	Member of the SWI/SNF family, transcription factor

*Mitochondrial components*				
(i) tRNAleu	mtDNA A3243G	Maternal	mtDNQ	Function unknown
(ii) Parahydroxybenzoate polyprenyltransferase	COQ2	AR	4q21 22	Functions in the final steps in the biosynthesis of CoQ
(iii) Coenzyme Q10 biosynthesis monooxygenase 6	COQ6	AR	14q24.3	Member of the ubiH/COQ6 family, required for the biosynthesis of coenzyme Q10
(iv) Decaprenyl diphosphate synthase subunit 2	PDSS2	AR	6q21	Enzyme that synthesizes the prenyl side chain of coenzyme Q or ubiquinone
(v) AarF domain-containing protein kinase 4	ADCK4	AR	19q13.2	Precise function unknown (possible protein kinase activity)

*Lysosomal proteins*				
(i) Lysosomal integral membrane protein type 2	SCARB2	AR	4q13 21	Type III glycoprotein located in limiting membranes of lysosomes and endosomes

*Unknown cellular location*				
(i) Apolipoprotein L1	APOL1	AR	22q12	Secreted high density lipoprotein, involved in the formation of cholesteryl esters and efflux of cholesterol from cells

## References

[1] Kitiyakara C., Kopp J. B., Eggers P. (2003). Trends in the epidemiology of focal segmental glomerulosclerosis. *Seminars in Nephrology*.

[2] Kitiyakara C., Eggers P., Kopp J. B. (2004). Twenty-one-year trend in ESRD due to focal segmental glomerulosclerosis in the United States. *American Journal of Kidney Diseases*.

[3] Haas M., Meehan S. M., Karrison T. G., Spargo B. H. (1997). Changing etiologies of unexplained adult nephrotic syndrome: a comparison of renal biopsy findings from 1976–1979 and 1995–1997. *American Journal of Kidney Diseases*.

[4] Korbet S. M., Genchi R. M., Borok R. Z., Schwartz M. M. (1996). The racial prevalence of glomerular lesions in nephrotic adults. *American Journal of Kidney Diseases*.

[5] Freedman B. I., Soucie J. M., Stone S. M., Pegram S. (1999). Familial clustering of end-stage renal disease in blacks with HIV- associated nephropathy. *American Journal of Kidney Diseases*.

[6] Rivera F., Lopez-Gomez J. M., Perez-Garcia R. (2004). Clinicopathologic correlations of renal pathology in Spain. *Kidney International*.

[7] Dragovic D., Rosenstock J. L., Wahl S. J., Panagopoulos G., DeVita M. V., Michelis M. F. (2005). Increasing incidence of focal segmental glomerulosclerosis and an examination of demographic patterns. *Clinical Nephrology*.

[8] Chun M. J., Korbet S. M., Schwartz M. M., Lewis E. J. (2004). Focal segmental glomerulosclerosis in nephrotic adults: presentation, prognosis, and response to therapy of the histologic variants. *Journal of the American Society of Nephrology*.

[9] Rydel J. J., Korbet S. M., Borok R. Z., Schwartz M. M. (1995). Focal segmental glomerular sclerosis in adults: presentation, course, and response to treatment. *American Journal of Kidney Diseases*.

[10] Kambham N., Markowitz G. S., Valeri A. M., Lin J., D'Agati V. D. (2001). Obesity-related glomerulopathy: an emerging epidemic. *Kidney International*.

[11] Praga M., Hernández E., Morales E. (2001). Clinical features and long-term outcome of obesity-associated focal segmental glomerulosclerosis. *Nephrology Dialysis Transplantation*.

[12] D'Agati V. D., Kaskel F. J., Falk R. J. (2011). Focal segmental glomerulosclerosis. *The New England Journal of Medicine*.

[13] Praga M., Morales E., Herrero J. C. (1999). Absence of hypoalbuminemia despite massive proteinuria in focal segmental glomerulosclerosis secondary to hyperfiltration. *American Journal of Kidney Diseases*.

[14] Praga M., Borstein B., Andres A. (1991). Nephrotic proteinuria without hypoalbuminemia: clinical characteristics and response to angiotensin-converting enzyme inhibition. *American Journal of Kidney Diseases*.

[15] Detwiler R. K., Falk R. J., Hogan S. L., Jennette J. C. (1994). Collapsing glomerulopathy: a clinically and pathologically distinct variant of focal segmental glomerulosclerosis. *Kidney International*.

[16] Laurinavicius A., Hurwitz S., Rennke H. G. (1999). Collapsing glomerulopathy in HIV and non-HIV patients: a clinicopathological and follow-up study. *Kidney International*.

[17] Ramsuran D., Bhimma R., Ramdial P. K. (2012). The spectrum of HIV-related nephropathy in children. *Pediatric Nephrology*.

[18] Hinkes B. G., Mucha B., Vlangos C. N. (2007). Nephrotic syndrome in the first year of life: two thirds of cases are caused by mutations in 4 genes (NPHS1, NPHS2, WT1, and LAMB2). *Pediatrics*.

[19] Santín S., Bullich G., Tazón-Vega B. (2011). Clinical utility of genetic testing in children and adults with steroid-resistant nephrotic syndrome. *Clinical Journal of the American Society of Nephrology*.

[20] Lipska B. S., Iatropoulos P., Maranta R. (2013). Genetic screening in adolescents with steroid-resistant nephrotic syndrome. *Kidney International*.

[21] D'Agati V. D., Fogo A. B., Bruijn J. A., Jennette J. C. (2004). Pathologic classification of focal segmental glomerulosclerosis: a working proposal. *American Journal of Kidney Diseases*.

[22] D'Agati V. D., Alster J. M., Jennette J. C. (2013). Association of histologic variants in FSGS clinical trial with presenting features and outcomes. *Clinical Journal of the American Society of Nephrology*.

[23] Stefanidis C. J., Querfeld U. (2011). The podocyte as a target: cyclosporin A in the management of the nephrotic syndrome caused by WT1 mutations. *European Journal of Pediatrics*.

[24] Weber S., Gribouval O., Esquivel E. L. (2004). NPHS2 mutation analysis shows genetic heterogeneity of steroid-resistant nephrotic syndrome and low post-transplant recurrence. *Kidney International*.

[25] Sprangers B., Kuypers D. R. (2013). Recurrence of glomerulonephritis after renal transplantation. *Transplantation Reviews*.

[26] Kriz W., Hähnel B., Hosser H. (2003). Pathways to recovery and loss of nephrons in anti-Thy-1 nephritis. *Journal of the American Society of Nephrology*.

[27] Remuzzi A., Pergolizzi R., Mauer M. S., Bertani T. (1990). Three-dimensional morphometric analysis of segmental glomerulosclerosis in the rat. *Kidney International*.

[28] Fuiano G., Comi N., Magri P. (1996). Serial morphometric analysis of sclerotic lesions in primary ‘focal’ segmental glomerulosclerosis. *Journal of the American Society of Nephrology*.

[29] Howie A. J., Pankhurst T., Sarioglu S., Turhan N., Adu D. (2005). Evolution of nephrotic-associated focal segmental glomerulosclerosis and relation to the glomerular tip lesion. *Kidney International*.

[30] Stokes M. B., Valeri A. M., Markowitz G. S., D'Agati V. D. (2006). Cellular focal segmental glomerulosclerosis: clinical and pathologic features. *Kidney International*.

[31] Thomas D. B., Franceschini N., Hogan S. L. (2006). Clinical and pathologic characteristics of focal segmental glomerulosclerosis pathologic variants. *Kidney International*.

[32] Gipson D. S., Trachtman H., Kaskel F. J. (2011). Clinical trial of focal segmental glomerulosclerosis in children and young adults. *Kidney International*.

[33] Deegens J. K. J., Dijkman H. B. P. M., Borm G. F. (2008). Podocyte foot process effacement as a diagnostic tool in focal segmental glomerulosclerosis. *Kidney International*.

[34] Markowitz G. S., Nasr S. H., Stokes M. B., D'Agati V. D. (2010). Treatment with IFN-*α*,-*β*,or-*γ* is associated with collapsing focal segmental glomerulosclerosis. *Clinical Journal of the American Society of Nephrology*.

[35] Markowitz G. S., Appel G. B., Fine P. L. (2001). Collapsing focal segmental glomerulosclerosis following treatment with high-dose pamidronate. *Journal of the American Society of Nephrology*.

[36] Wei C., Möller C. C., Altintas M. M. (2008). Modification of kidney barrier function by the urokinase receptor. *Nature Medicine*.

[37] Wei C., Trachtman H., Li J. (2012). Circulating suPAR in two cohorts of primary FSGS. *Journal of the American Society of Nephrology*.

[38] Li F., Zheng C., Zhong Y. (2014). Relationship between serum soluble urokinase plasminogen activator receptor level and steroid responsiveness in FSGS. *Clinical Journal of the American Society of Nephrology*.

[39] Wada T., Nangaku M., Maruyama S. (2014). A multicenter cross-sectional study of circulating soluble urokinase receptor in Japanese patients with glomerular disease. *Kidney International*.

[40] Maas R. J. H., Wetzels J. F. M., Deegens J. K. J. (2012). Serum-soluble urokinase receptor concentration in primary FSGS. *Kidney International*.

[41] Meijers B., Maas R. J. H., Sprangers B. (2014). The soluble urokinase receptor is not a clinical marker for focal segmental glomerulosclerosis. *Kidney International*.

[42] Bock M. E., Price H. E., Gallon L., Langman C. B. (2013). Serum soluble urokinase-type plasminogen activator receptor levels and idiopathic FSGS in children: a single-center report. *Clinical Journal of the American Society of Nephrology*.

[43] Spinale J. M., Mariani L. H., Kapoor S. (2015). A reassessment of soluble urokinase-type plasminogen activator receptor in glomerular disease. *Kidney International*.

[44] Sinha A., Bajpai J., Saini S. (2014). Serum-soluble urokinase receptor levels do not distinguish focal segmental glomerulosclerosis from other causes of nephrotic syndrome in children. *Kidney International*.

[45] Meijers B., Sprangers B. (2014). The hype cycle for soluble urokinase receptor in fsgs: passing the trough of disillusionment?. *Clinical Journal of the American Society of Nephrology*.

[46] Franco Palacios C. R., Lieske J. C., Wadei H. M. (2013). Urine but not serum soluble urokinase receptor (suPAR) may identify cases of recurrent FSGS in kidney transplant candidates. *Transplantation*.

[47] Hayek S. S., Sever S., Ko Y.-A. (2015). Soluble urokinase receptor and chronic kidney disease. *The New England Journal of Medicine*.

[48] Reiser J., Von Gersdorff G., Loos M. (2004). Induction of B7-1 in podocytes is associated with nephrotic syndrome. *Journal of Clinical Investigation*.

[49] Yu C.-C., Fornoni A., Weins A. (2013). Abatacept in B7-1-positive proteinuric kidney disease. *The New England Journal of Medicine*.

[50] Garin E. H., Diaz L. N., Mu W. (2009). Urinary CD80 excretion increases in idiopathic minimal-change disease. *Journal of the American Society of Nephrology*.

[51] Garin E. H., Mu W., Arthur J. M. (2010). Urinary CD80 is elevated in minimal change disease but not in focal segmental glomerulosclerosis. *Kidney International*.

[52] Cara-Fuentes G., Wei C., Segarra A. (2014). CD80 and suPAR in patients with minimal change disease and focal segmental glomerulosclerosis: diagnostic and pathogenic significance. *Pediatric Nephrology*.

[53] Benigni A., Gagliardini E., Remuzzi G. (2014). Abatacept in B7-1-positive proteinuric kidney disease. *The New England Journal of Medicine*.

[54] Alachkar N., Carter-Monroe N., Reiser J. (2014). Abatacept in B7-1-positive proteinuric kidney disease. *The New England Journal of Medicine*.

[55] Fiorina P., Vergani A., Bassi R. (2014). Role of podocyte B7-1 in diabetic nephropathy. *Journal of the American Society of Nephrology*.

[56] Gagliardini E., Novelli R., Corna D. (2016). B7-1 is not induced in podocytes of human and experimental diabetic nephropathy. *Journal of the American Society of Nephrology*.

[57] Smeets B., Stucker F., Wetzels J. (2014). Detection of activated parietal epithelial cells on the glomerular tuft distinguishes early focal segmental glomerulosclerosis from minimal change disease. *American Journal of Pathology*.

[58] Kuppe C., Gröne H.-J., Ostendorf T. (2015). Common histological patterns in glomerular epithelial cells in secondary focal segmental glomerulosclerosis. *Kidney International*.

[59] Fervenza F. C. (2013). A patient with nephrotic-range proteinuria and focal global glomerulosclerosis. *Clinical Journal of the American Society of Nephrology*.

[60] Grgic I., Campanholle G., Bijol V. (2012). Targeted proximal tubule injury triggers interstitial fibrosis and glomerulosclerosis. *Kidney International*.

[61] Sethi S., Glassock R. J., Fervenza F. C. (2015). Focal segmental glomerulosclerosis: towards a better understanding for the practicing nephrologist. *Nephrology Dialysis Transplantation*.

[62] Zenker M., MacHuca E., Antignac C. (2009). Genetics of nephrotic syndrome: new insights into molecules acting at the glomerular filtration barrier. *Journal of Molecular Medicine*.

[63] Löwik M. M., Groenen P. J., Levtchenko E. N., Monnens L. A., Van Den Heuvel L. P. (2009). Molecular genetic analysis of podocyte genes in focal segmental glomerulosclerosis—a review. *European Journal of Pediatrics*.

[64] Caridi G., Bertelli R., Carrea A. (2001). Prevalence, genetics, and clinical features of patients carrying podocin mutations in steroid-resistant nonfamilial focal segmental glomerulosclerosis. *Journal of the American Society of Nephrology*.

[65] Ruf R. G., Lichtenberger A., Karle S. M. (2004). Patients with mutations in NPHS2 (podocin) do not respond to standard steroid treatment of nephrotic syndrome. *Journal of the American Society of Nephrology*.

[66] Bullich G., Trujillano D., Santín S. (2015). Targeted next-generation sequencing in steroid-resistant nephrotic syndrome: mutations in multiple glomerular genes may influence disease severity. *European Journal of Human Genetics*.

[67] Barua M., Brown E. J., Charoonratana V. T., Genovese G., Sun H., Pollak M. R. (2013). Mutations in the INF2 gene account for a significant proportion of familial but not sporadic focal and segmental glomerulosclerosis. *Kidney International*.

[68] Kopp J. B., Smith M. W., Nelson G. W. (2008). MYH9 is a major-effect risk gene for focal segmental glomerulosclerosis. *Nature Genetics*.

[69] Kao W. H. L., Klag M. J., Meoni L. A. (2008). MYH9 is associated with nondiabetic end-stage renal disease in African Americans. *Nature Genetics*.

[70] Genovese G., Friedman D. J., Ross M. D. (2010). Association of trypanolytic ApoL1 variants with kidney disease in African Americans. *Science*.

[71] Kopp J. B., Nelson G. W., Sampath K. (2011). APOL1 genetic variants in focal segmental glomerulosclerosis and HIV-associated nephropathy. *Journal of the American Society of Nephrology*.

[72] Kopp J. B., Winkler C. A., Zhao X. (2015). Clinical features and histology of apolipoprotein L1-associated nephropathy in the FSGS Clinical Trial. *Journal of the American Society of Nephrology*.

[73] Ko W. Y., Rajan P., Gomez F. (2013). Identifying Darwinian selection acting on different human APOL1 variants among diverse African populations. *The American Journal of Human Genetics*.

[74] Uzureau P., Uzureau S., Lecordier L. (2013). Mechanism of *Trypanosoma brucei* gambiense resistance to human serum. *Nature*.

